# Prognostic Significance of CDK1 in Ovarian and Cervical Cancers

**DOI:** 10.7150/jca.104371

**Published:** 2025-02-10

**Authors:** Cong Xu, Chaowen Chen, Yonghong Xu, Zhenqi Li, Huaqiu Chen, Guangming Wang

**Affiliations:** 1 School of Clinical Medicine, Dali University, Dali 671000, Yunnan, People's Republic of China.; 2 Chinese People's Life Safety Research Institute, Huaxi Hospital, Sichuan University, Chengdu 610000, Sichuan.; 3 Chongqing Medical University, Chongqing, Banan 401300, People's Republic of China.; 4 West China School of Medicine, Sichuan University, Chengdu, People's Republic of China.; 5 Department of Clinical Laboratory, Xichang People's Hospital, Xichang, People's Republic of China.; 6 Center of Genetic Testing, Dali University, Dali 671000, People's Republic of China.

**Keywords:** CDK1, ovarian cancer, cervical cancer, prognostic marker

## Abstract

**Background:** Ovarian cancer (OC) and cervical cancer (CC) are the leading causes of death among women. Therefore, identifying markers for early detection and treatment is critical. CDK1 governs the G2/M transition of the cell cycle and is a significant regulatory protein of the cycle. RO-3306 and UBE2C are related to CDK1 expression and might jointly facilitate the development of OC. CDK1 and CDK2 phosphorylate MLK3, which plays an important role in the invasion and proliferation of OC cells. Furthermore, miR-490-3P targets CDK1 and restrains the growth of ovarian tumors. CDK1 also plays a crucial part in the progression of CC. For instance, CDK1 overexpression can rescue the effect of RCC1 knockdown, which is involved in key processes, such as cytoplasmic transport, on G1 cell cycle progression. Using bioinformatics analysis, we evaluated the functional enrichment and role of the co-expressed gene CDK1 in these two cancers and its impact on their prognoses.

**Methods:** First, we screened public datasets for OC- and CC-associated DEGs and identified intersecting genes. Enrichment analyses of these genes revealed key biological pathways and processes. We then generated protein-protein interaction networks to identify central genes and important gene modules.

**Results:** Additional enrichment analyses revealed that cell cycle regulation and germ cell maturation were the primary processes regulated by these core genes. We also examined the function of CDK1 in OC and CC, demonstrating its overexpression and its association with particular immunological cell infiltration patterns. Furthermore, CDK1 mutational burden, copy number variation, and patient survival analyses indicated that CDK1 may be a useful prognostic marker. Finally, immunohistochemical examination confirmed the expression of some candidate genes in clinical samples.

**Conclusion:** These findings shed light on the molecular causes of OC and CC and will aid the identification of novel targets for future research regarding these cancers, including their diagnosis and treatment.

## Background

Middle-aged women are disproportionately affected by cervical cancer (CC), which is the fourth most common malignancy worldwide among women 1. The primary cause of cervical lesions is infection with the high-risk human papillomavirus (HR-HPV), particularly HR-HPV strains 16 and 18. Persistent cervical HR-HPV infection can progress to invasive CC 2. Approximately 600,000 women—particularly middle-aged women and those living in underdeveloped areas—are affected by CC annually. Although widespread HPV screening and vaccination can avert most CC-related fatalities, early detection and diagnosis are crucial for CC management. The choice of appropriate and precise therapeutic targets can notably impact treatment results.

In addition to being a primary cause of cancer-related deaths among women worldwide, ovarian cancer (OC) also has a dismal 5-year survival rate of approximately 49%. Much interest has been paid to alternative treatments because of the poor therapeutic responses of OC to traditional treatments, such as bevacizumab and poly (ADP-ribose) polymerase inhibitors, which target specific molecules 3. OC exhibits increased recurrence and symptomatic stress compared with other cancer types. It is also linked to sexual dysfunction and a marked decrease in the quality of life of patients 4. Cyclin-dependent kinase 1 (CDK1) exhibits serine/threonine protein kinase activity and has numerous roles in the cell, including apoptosis, gene expression regulation, and cell cycle control 5. Among various tumor-supporting factors, CDK is considered by some scholars to be a novel and worthy candidate for further study in cancer therapy and plays a key role in the cell cycle process 6. CDK1 can be considered an optimal CDK target for breast cancer treatment, promoting MYC-driven transcription and tumorigenesis through mediated ubiquitin ligase activation of FBXO28 and predicting poor survival in breast cancer. In addition, in clinical applications, when CDK1 is combined with flavopiridol and paclitaxel to treat patients with esophageal, lung, and prostate cancers, toxicity is manageable and clinical activity can be observed 7.

Most members of the CDK family are linked to survival rates for a variety of cancer types and show significantly higher expression in cancer tissues than in normal tissues. Moreover, tumorigenesis is closely associated with CDK1 dysregulation 8. However, although there have been some articles related to the study of CDK1 in CC and OC, there is still a deficiency of in-depth discussion regarding the role of CDK1 as a co-expressed gene in the clinical prognosis of the two cancers.

Whereas pathological diagnosis is considered the gold standard for detecting CC and OC, pathological investigation alone cannot aid in identifying the early stages of cell morphological changes. These findings in early CC and OC testing may not always indicate abnormalities 9. Although CDK1 may be a promising target for cancer therapy, its mechanisms and importance in carcinogenesis and cancer treatment are unelucidated. Consequently, in this study, we employed bioinformatics analysis to explore the functional enrichment and immune infiltration of the co-expressed gene CDK1 in OC and CC, as well as its role in clinical diagnosis and treatment.

## Materials and Methods

### Gene set variation analysis scores, differential expression of CDK1 in CC and OC, and immune cell correlation analysis

The gene set variation analysis (GSVA) score represents the change in gene set activity in a specific cancer sample population in an unsupervised manner and positively correlates with gene set expression. This study used the Gene Set Cancer Analysis (GSCA) database (http://guolab.wchscu.cn) to analyze the GSVA scores for CDK1 in CC and OC. Various immune cells play different roles in tumor formation. Therefore, investigation of the mechanisms of tumor formation and treatment often requires in-depth quantitative studies on the immune infiltration status of different tumor types. Thus, this study analyzed CDK1-associated lymphocytes using data from the GSCA website.

### Gene mutation load, copy number and survival analyses, and establishment of the core gene prediction model

Using the cBioPortal database (https://www.cbioportal.org/) and the GSCA database (http://guolab.wchscu.cn), we analyzed the CDK1 mutation loads in CC and OC. We also analyzed the impact of CDK1 copy number on survival in patients with CC and OC and plotted survival curves. Multivariate Cox proportional risk regression analysis was performed using the candidate prognostic genes to construct a prognostic risk model. Next, CC-related data from The Cancer Genome Atlas (TCGA) database and OC-related data from the International Cancer Genome Consortium (ICGC) database were used as training datasets and assessed separately to evaluate the predictive accuracy of the prognostic risk model. Statistical significance was set at p < 0.05 10.

We then divided the patient population into low- and high-risk categories based on the median risk score. The “Survival” toolkit in R was used to conduct a Kaplan-Meier survival analysis in these groups to evaluate the risk sensitivity and uniqueness. Variations in the expression of these well-known genes in healthy versus cancerous tissues were mapped using R tools, including reshape2 and BiocManager 11. Next, receiver operating characteristic curves (ROC) curves were constructed using the “timeROC” package in R. The common clinical disorders were categorized and forecasted, and the outcomes were presented. The predictive power of the nomogram was assessed based on the area under the curve (AUC) values.

### Clinical sample information and immunohistochemistry

To assess differences in CDK1 in cervical and ovarian cancer, we utilized OC microarray (F100Ov02, Bioaitech Co., Ltd.), which encompassed 90 serous tumors and 10 ovarian cancer adjacent tissue. The average age of patients who provided ovarian cancer adjacent tissue was 51 years old, and it included stage III-IV OC tissues. The clinical information parameters of patients with ovarian cancer are described in detail in Table 1. Thirty cases of cervical squamous cell carcinoma and 30 cases of paracancer tissue were included in the CC chip (IWLT-N-60CC31, Servicebio) for immunohistochemistry investigation, and the average age of the person donating the tissue was 52 years old. The clinical parameters of the chip are described in detail in Table 2.

#### Inclusion and exclusion criteria for patients with CC

Inclusion criteria: (1) Age ranging from 35 to 65 years. (2) Diagnosis of cervical cancer through cervical biopsy. (3) Cervical squamous cell carcinoma, confirmed histologically. (3) Adhere to the Cervical Cancer Diagnosis and Treatment Guidelines (4) Not pregnant or breastfeeding. (5) All patients provide written informed consent before enrollment and before the start of any procedure.

Exclusion Criteria: (1) Cervical cancer complicated with other malignant tumors. (2) Acute infection or uncontrolled serious medical conditions. (3) Pregnancy or lactation. (4) Myocardial dysfunction, defined as NYHA grade >2 (5) Loss of follow-up. (6) Failure to meet the inclusion criteria.

#### Inclusion and exclusion criteria for patients with OC

Inclusion Criteria: (1) Age ranging from 30 to 80 years. (2) Histologically confirmed diagnosis of ovarian cancer. (3) Ovarian cancer stages are classified according to TNM, including stage IIIA-VI. (4) Not pregnant or breastfeeding. (5) Comply with the Ovarian Cancer Guidelines (2022 edition). (6) All participants provide written informed consent.

Exclusion Criteria: (1) Cardiovascular disease, diabetes, acute inflammation, blood diseases, kidney disease, or other cancers. (3) Failure to meet the inclusion criteria.

The expression of candidate genes in both the illness group and the control group was validated by immunohistochemical analysis. The paraffin sections were placed in the environmentally friendly dewaxing solution and anhydrous ethanol for 10 minutes each, and then rinsed with distilled water. Subsequently, antigen repair was conducted to prevent excessive evaporation of the buffer, which was washed with PBS after natural cooling. Next, the endogenous peroxidase was blocked, the serum was enclosed, and the sections were incubated overnight with a single antibody. The second antibody was added and incubated for 50 minutes. After DAB color development, the nucleus was restained. Finally, the sections were dehydrated and sealed, and the results were interpreted under a white light microscope. The antibodies used in this study were as follows: CDK1 (PTGLAB, 1:200), and the second antibody utilized was goat anti-Rabbit IgG labeled by HRP (Servicebio, 1:200).

### Protein-protein interaction network, Gene Ontology, and Kyoto Encyclopedia of Genes and Genomes enrichment analyses for CDK1

STRING is an online-based protein-protein interaction (PPI) tool used to build PPI networks with recognized differentially expressed genes (DEGs). We used this website to identify genes closely associated with CAV1 and to map the PPI network.

Additionally, we used the INPUT 2.0 database (http://metascape.org) for gene ontology (GO) and Kyoto Encyclopedia of Genes and Genomes (KEGG) analysis. The “clusterProfiler,” “tringi,” and “colorspace” packages in R were used to import the intersection gene visualization into the Metascape database 12.

### Statistical analysis

Student's t-test was utilized to ascertain the significant DEGs between the tumor and non-tumor samples. One-way analysis of variance was employed to examine variances across three or more groups. Kaplan-Meier analysis using the log-rank test was employed to compare the survival rates in the different groups. All data analysis in this study was performed utilizing R (4.4.1). The R software tools BiocManager, limma, Survival, Survminer, and GSVA were used to analyze gene expression differences, survival rates, and immune cell correlations in CC tissues. In the Cox regression analysis, R-package limma, gmnet, survival, and Survminer were employed to identify immune genes linked to CC prognosis; P-values lesser than 0.05 were considered to indicate statistical significance.

### Mendelian randomization study

Single nucleotide polymorphisms (SNPs) were used as instrumental variables (IV) to assess the causal relationship between CDK1 and CC and between CDK1 and OC. For SNPS related to CC and OC, we chose P < 5e-08 as a strict criterion for genome-wide significance to extract exposure data. The analysis is based on three assumptions: (1) IVs are closely related to exposure; (2) IVs are not associated with potential confounders; (3) IVs do not directly affect results except through exposure. The random effects inverse Variance Weighting (IVW-RE) model is used as the primary analytical method to examine causality. The weighted mode method evaluates the impact of different genotypes on the phenotype by calculating the weighted average of each genotype. MR-Egger avoids forcing the regression line through the origin, allowing for directional genetic pleiotropy in the IVs involved. The term “simple mode” typically denotes fundamental statistical frameworks that avoid complex modifications or numerous variables yet are employed in initial analyses to evaluate variables with direct correlations. Sensitivity analysis was conducted to assess the robustness of our findings, eliminating bias and determining the impact of IVs on outcome variables. The “MR-PRESSO” package was used to identify and correct horizontal pleiotropy. Cochran's Q test assessed SNP estimate heterogeneity; a P-value <0.05 indicated no heterogeneity, suggesting the fixed-effect IVW technique as the primary method. Cochran's Q value (P < 0.05) identified heterogeneity, and MR-Egger analysis detected horizontal pleiotropy 13.

## Results

### Differential expression of CDK1 in CC and OC and immunohistochemical results

**Figure 1:**
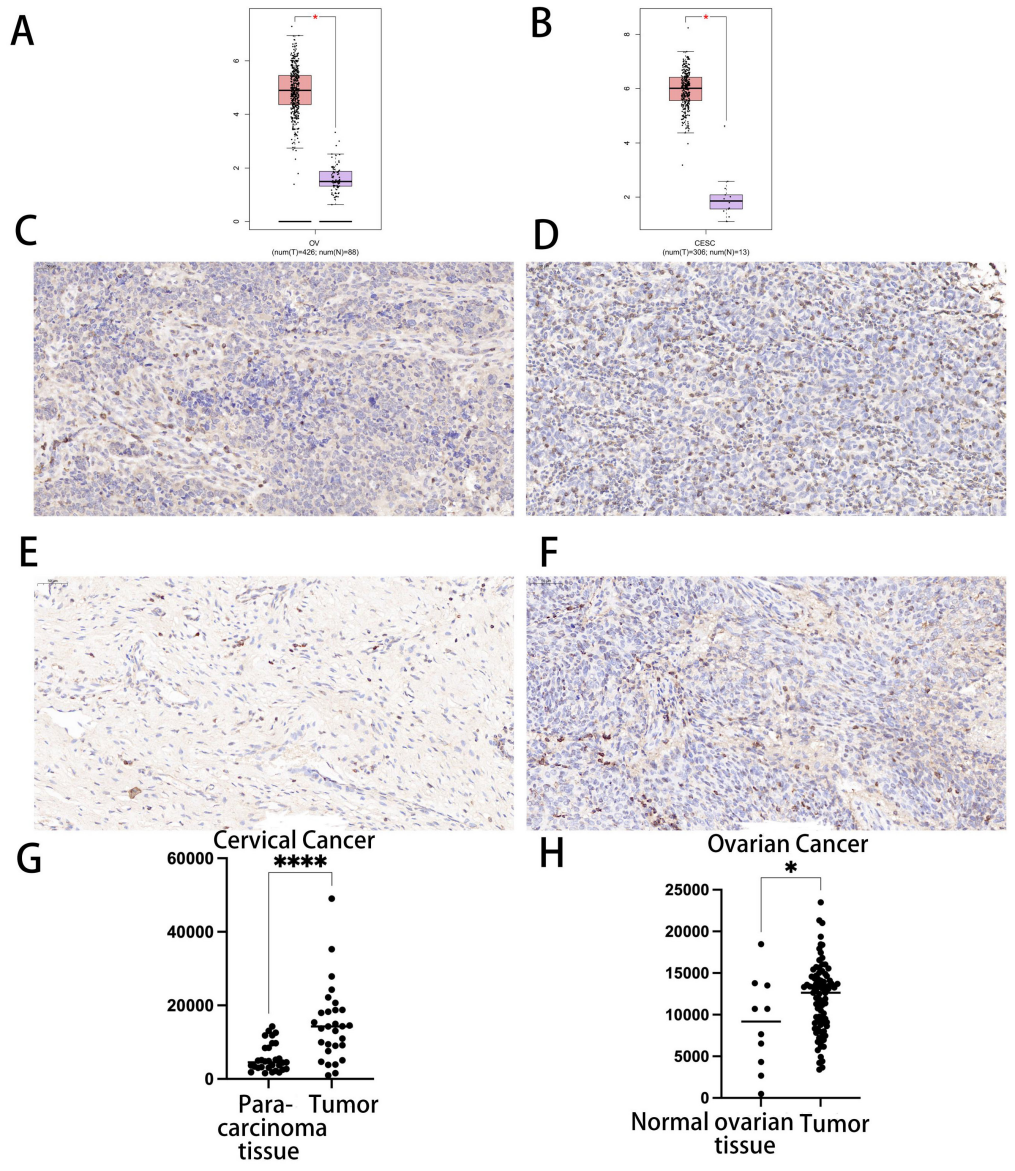
Differential expression of CDK1 in cervical cancer and ovarian cancer and immunohistochemical analysis. (A) Differential expression of CDK1 in cervical cancer. (B) Differential expression of CDK1 in ovarian cancer. (C) Immunohistochemical analysis of CDK1 expression in cervical cancer tissues. (D) Immunohistochemical analysis of CDK1 expression in normal cervical tissues. (E) Immunohistochemical analysis of CDK1 expression in ovarian cancer. (F) Immunohistochemical analysis of CDK1 expression in normal ovarian tissue. (G) Comparison of IOD between cervical cancer tissue and normal cervical tissue. (H) Comparison of IOD between ovarian cancer and normal ovarian tissue (p < 0.05). IOD, integrated optical density.

The transcript levels of CDK1 in CC and OC were analyzed in two datasets from the TCGA database. In both datasets, CDK1 expression levels in CC and CC tissues were significantly upregulated compared with those in normal tissues (Fig. 1 A, B).

We treated cancer tissue and normal organ tissue with immunohistochemistry (IHC) to confirm the expression of CDK1 in CC tissue and normal cervical tissue, as well as CDK in OC tissue and normal ovarian tissue. We discovered that CDK1 was substantially expressed in cancer tissue as compared to the control group. In line with our bioinformatics prediction, there was a statistically significant link between CDK1 in groups of patients with CC and OC and normal tissues (p < 0.05) (Fig. 1C-1H).

### Immune cell associations in OC and CC

**Figure 2:**
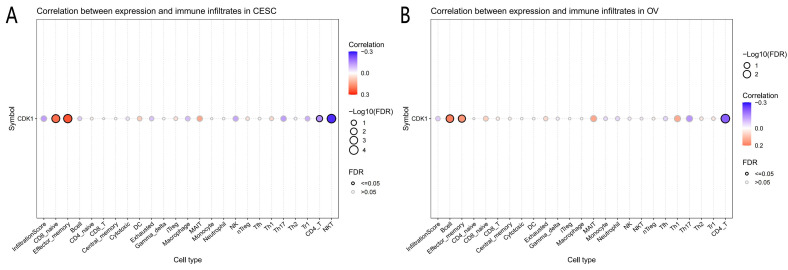
Analysis results of correlation between genes and immunoinfiltrating cells. (A) Association between immunoinfiltrating cells with CDK1 in cervical cancer. (B) Association between immunoinfiltrating cells with CDK1 in ovarian cancer.

Analysis of immune cell infiltration showed that CDK1 expression was significantly correlated with CD8 naïve cells, effector memory cells, CD4 T cells, and natural killer T (NKT) cells in CC. Similarly, CDK1 was significantly associated with B cells, effector memory cells, and CD4 + T cells in OC (Fig. 2 A, B).

### Copy number variation (CNV), mutation load, GSVA scores, and survival analysis of the impact of CDK1 in CC and OC

**Figure 3:**
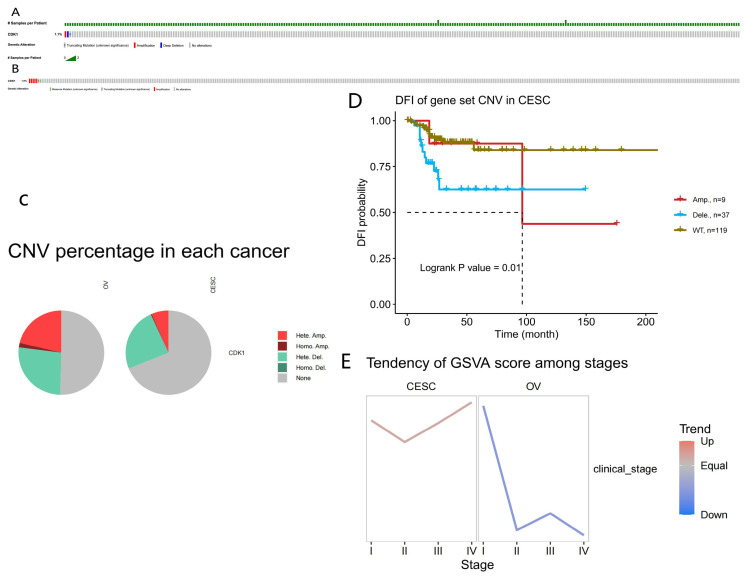
Prognostic value of copy number variation and tumor mutation load. (A) Influence of CDK1 mutation load in cervical cancer. (B) Mutational load of CDK1 in ovarian cancer. (C) CDK1 copy number ratio in cervical and ovarian cancer. (D) Correlation between CDK1 copy number and Kaplan-Meier survival curve of cervical cancer. (E) GSVA scores for cervical and ovarian cancer at different stages.

The analysis of mutation load revealed that the proportion of CDK1 mutations in OC was 1%, which was relatively amplified, with a small number of proportional missense and truncating mutations. The proportion of CDK1 mutations in CC was 1.8%, including deep deletions and amplifications, which was a large proportion. A small number were truncating mutations (Fig. 3A, B). Figure 2 depicts the distribution of CDK1 copy number variation (CNVs) in CC and OC, color-coded by CNV type (Fig. 3C).

In addition, this study analyzed the effects of DNA methylation of CDK1 on overall survival (OS), disease-specific survival (DSS), disease-free interval (DFI), and progression-free survival (PFS). The Kaplan-Meier survival curves demonstrated no association between CDK1 CNVs with poor prognosis in CC. The decrease in the number of CDK1 CNVs in CC tissues represents a poor prognosis and is a good prognostic indicator of DFI 14. DFI is a significant indicator of clinical benefit; if no recurrence or death is expected, the clinical benefit can be expressed more accurately, and the follow-up period can be shortened by adding the node of disease recurrence (Fig. 3D). The relationship between the GSVA score and the grading of CC and OC was also evaluated. Patients with high GSVA scores had worse outcomes than those with low GSVA scores. The GSVA score of CDK1 associated with CC and OC represented the comprehensive level of gene set expression and was positively correlated with gene expression (Fig. 3E).

**Figure 4:**
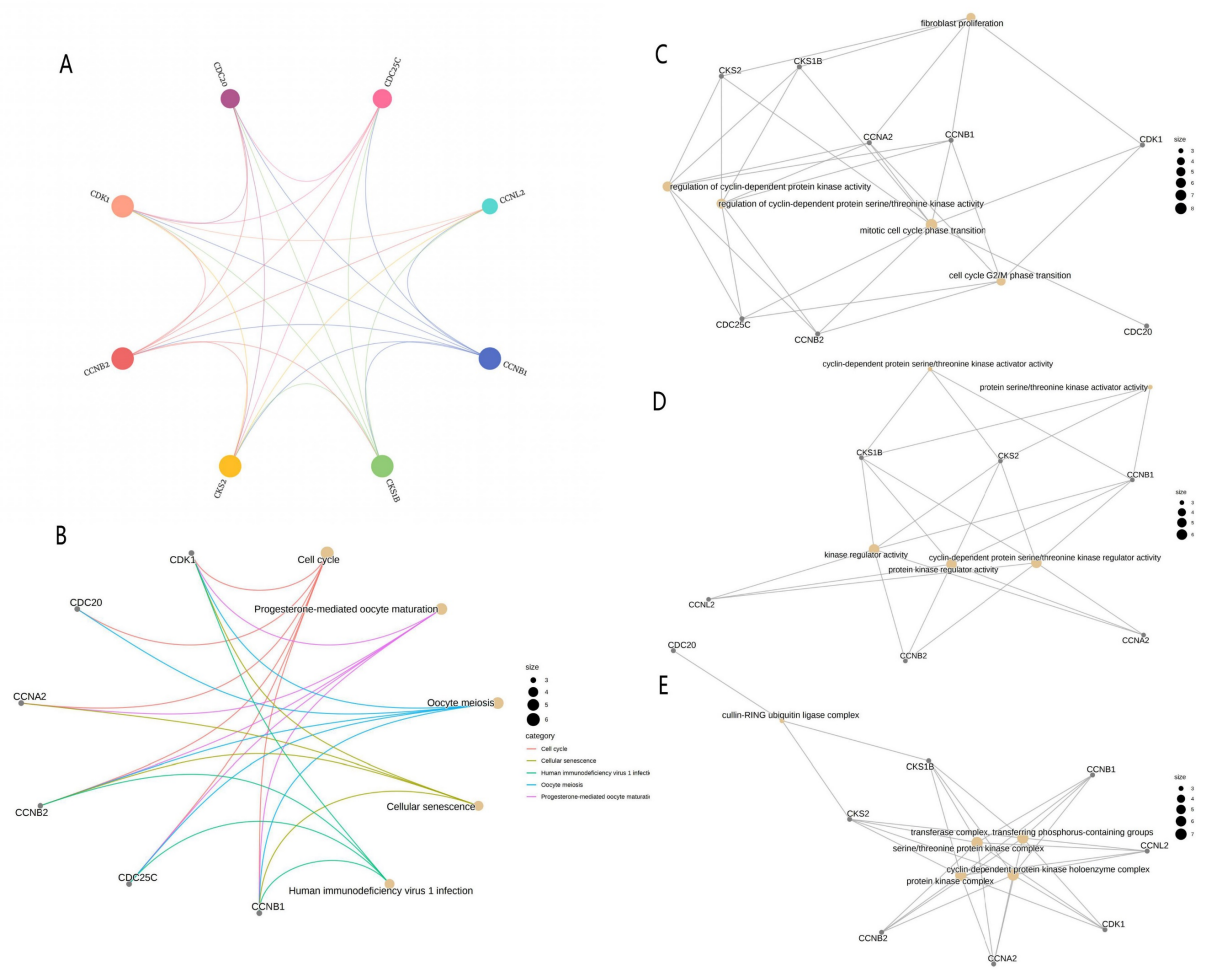
Diagnostic model of CDK1 in cervical and ovarian cancer. (A) Grouping of high-risk and low-risk patients with ovarian cancer. (B) Survival status of patients with ovarian cancer with different risk scores. (C) Expression heat maps of prognostic related genes in high- and low-risk groups of ovarian cancer. (D) Time-dependent receiver operating characteristic (ROC) analysis curve of CDK1 in ovarian cancer. (E) Time-dependent ROC analysis curve of CDK1 in cervical cancer. (F) Grouping of high-risk and low-risk patients with cervical cancer. (G) Survival status of patients with cervical cancer with different risk scores. (H) Heat maps of prognostic related genes in high-risk and low-risk groups of cervical cancer.

The median RiskScore was used to classify each patient's TCGA-CC and TCGA-OC data into low or high RiskScore groups, as illustrated in image 10. Individuals with higher CDK1 expression levels had a higher chance of survival. Additionally, the sensitivity and specificity of the OS model were confirmed using receiver operating characteristic (ROC) curve analysis (Fig. 4) 15. The area under the ROC curve (AUC) values for CC and OC outcomes over 1, 3, and 5 years, as predicted by the current risk models, were 0.517, 0.49, and 0.529, respectively, and 0.507, 0.511, and 0.5, respectively; thus, the model's accurate prediction of survival rates in CC and OC patients was validated despite the negligible role of CDK1 in OS survival trials.

### PPI analysis and analysis of differential gene enrichment

**Figure 5:**
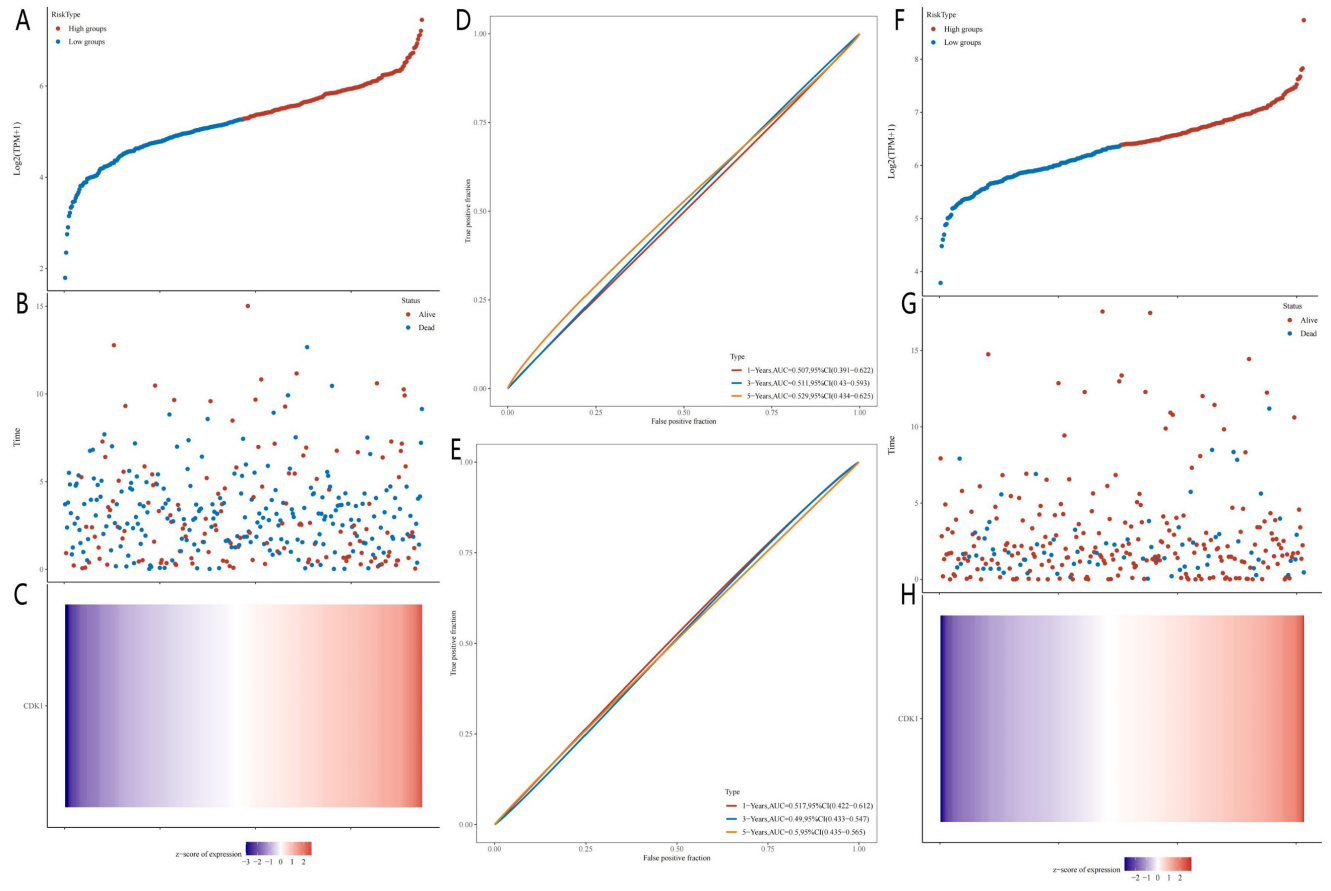
** PPI network and enrichment analysis**. (A) PPI network composed of CDK1-related genes. (B) KEGG functional enrichment analysis for grading the differentially expressed genes. GO functional enrichment analysis of differential genes included the analyses of the genes enriched in (C) the biological process, (D) the molecular function, and (E) cellular component categories. PPI, protein-protein interaction; GO, Gene Ontology; KEGG, Kyoto Encyclopedia of Genes and Genomes; BP, biological process; MF, molecular function; CC, cell component.

A total of 10 linked genes of CDK1 were analyzed using the STRING website (Fig. 5A), including CCNA1, CCNB1, CCNL2, CKS2, CKS1B, CDC25C, CCNB2, CCNA2, CDC20, and BUB1B. Through the study of the biological role of hub genes, these targets are highly enriched in biological processes, cell components, molecular functional categories, and KEGG pathways. Terms enriched in the categories of biological processes, molecular functions, and cell components, Including “transferase complex, transferring phosphorus-containing groups,” “regulation of cyclin-dependent protein serine/threonine kinase activity,” “mitotic cell cycle phase transition,” “fibroblast proliferation,” “cell cycle G2/M phase transition,” “transferase complex, transferring phosphorus-containing groups,” and “cyclin-dependent protein serine/threonine kinase activator activity.” Many of these effects are related to biological activities such as gene transcription and cycle. KEGG pathway analysis revealed that these genes are related to “Progesterone-mediated oocyte maturation” and “Cellular senescence.” There is a close relationship between “Human immunodeficiency virus 1 infection” and “Oocyte meiosis” (Fig. 5B-5E).

### Results of Mendelian randomization

The combined results of Mendelian randomization analysis showed that CDK1 (P=0.02, odds ratio [OR], 1.001 [95% CI, 1.000-1.003]; In OC, CDK1 (P=0.03; OR, 1.0.782 [95% CI, 0.619-0.988]). These results indicate that CDK1 is significantly correlated with CC and OC, and the expression of CDK1 is positively correlated with its occurrence and development in CC, whereas the expression of CDK1 is negatively correlated with its occurrence and development in OC (Table 3).

## Discussion

OC and CC are major global health hazards in women with cancer. These cancers not only contribute to the increase in cancer cases among women but are also the primary cause of cancer-related mortality in women 16,17. Despite significant research efforts, the association between gene mutation and treatment for OC and CC remains controversial 18. The complex genetic basis of cancer is not fully explained by current data, suggesting unknown genetic factors in its development.

Consequently, this study aimed to evaluate the risks of OC and CC development by searching for novel core-targeted genes to provide additional therapeutic avenues for treating these cancers 19. Few reliable biomarkers exist for OC and CC detection and diagnosis. For patients at a high risk of developing cancer, finding and identifying novel biomarkers is crucial for early screening and diagnosis 20. Furthermore, before determining a course of treatment or surgical plan, the final pathological diagnosis and staging are crucial due to the limitations of clinical staging. Improving individualized therapy requires additional research on the predicted biological indicators of CC and OC to help classify patients with cancer as low or high risk before surgery 21.

Previous studies have indicated that CDK1 plays a role in both CC and OC. CDK1 is a crucial for regulating the cell cycle by controlling the transitions of the G2/M phase. RO-3306, a CDK1 inhibitor, reduces cell proliferation and tumor growth, induces apoptosis, causes cell stress, and inhibits cell migration 22.

Thus, it provides biological evidence for clinical trials of CDK1 inhibitors in OC. Additionally, UBE2C expression is highly correlated with CDK1 expression in OC tissues and cell lines, suggesting that UBE2C might cooperate with CDK1 in ovarian tumorigenesis 23.

Furthermore, MLK3 is a serine/threonine mitogen-activated protein kinase that is essential for the invasion and proliferation of OC cells. The phosphorylation of MLK3 by CDK1 and CDK2 is significant for the division of OC cells 24. The overexpression of miR-490-3P leads to cell cycle arrest and apoptosis and reduces cell proliferation, migration, and invasion because it directly targets CDK1. *In vivo* studies have demonstrated that miR-490-3P can inhibit tumor development; decrease the expression of CDK1, Bcl-xL, and MMP2/9; and increase P53 expression. This implies that miR-490-3P may suppress the development of ovarian epithelial cancer by targeting CDK1^25^. Part of the role of CDK1 in CC has also been verified accordingly. RCC1 is the main guanine nucleotide exchange factor of Ran GTPase and plays a key role in nucleoplasmic transport, mitosis, and nuclear membrane assembly. A study found that CDK1 is involved in the G1 checkpoint regulation in CC cells. CDK1 knockdown can inhibit G1/S progression, while its overexpression can reverse the effect of RCC1 knockdown on G1 cell cycle progression. Additionally, CDK1 is frequently observed in KEGG pathways, including oocyte meiosis, cell cycle, and progestogen-mediated oocyte maturation, suggesting that it plays an important role in the progression of CC 26, 27.

To better understand the molecular mechanism of CDK1 in the carcinogenesis and development of CC and OC, we performed GO and KEGG functional enrichment analysis of CDK1 and its related genes. These key genes were found to be involved in “Cell Cycle,” “Cellular senescence,” “Oocyte meiosis,” “Regulation of cyclin-dependent protein serine/threonine,” “kinase activity,” and “protein serine/threonine kinase activator activity.” According to KEGG pathway analysis, CDK1 and its related genes are closely related to “Progesterone-mediated oocyte maturation.” Protein kinase I (PGG-I) is dependent on cyclic guanosine phosphate (cGMP) and is a member of the serine/threonine kinase family that is important for tumor development. The cGMP/PKG I pathway is essential for the development and survival of human OC cells, and cGMP analogs can lead to growth restriction and death of a variety of cancer cells 28. Serine and threonine also play important roles in CC. A member of the serine/threonine kinase mixed lineage kinase (MLK) family is mixed lineage kinase 4 (MLK4). After confirming MLK4 expression in patients with cervical squamous cell carcinoma, we found that MLK4 was highly correlated with WHO typing and significantly overexpressed in CESC 29). Finally, compared with normal paracancer tissues, CC and OC showed increased CDK1 expression. The GSVA scores, degree of immune cell infiltration, and number of CNVs also showed correlations. Thus, CDK1 might be a helpful molecular marker for the diagnosis of both types of cancer and can also help predict the prognosis of patients with cancer. Furthermore, the predicted AUC values for 1-, 3-, and 5-year outcomes for clinical samples of CC and OC suggest that CDK1 may be involved in the treatment responses of CC and OC 30,31.

CDKs belong to the serine/threonine kinase family and are potential targets for cancer therapy. Members of this protein family have many cell phenotypes and tumor-specific antigen molecules with different functions. These proteins and cyclin complexes play crucial roles in all phases of the cell cycle. Changes in enzyme activity have also been observed in many different cell types and tumors 32.

Most CDKs show significantly higher expression levels in cancer tissues than in normal tissues and, according to the TCGA database, are associated with survival in multiple cancer types. CDK1 imbalances are closely related to tumor formation 33. Furthermore, CDK1 plays a role in regulating immune tolerance, tumor spread, and cell growth and diversification. Numerous cancer types are linked to CDK1 activation, and CDK1 phosphorylation across different substrates significantly hinders their roles in tumor formation 34. Further, we explored how the CDK1 gene affects the infiltration pattern of tumor immune cells and its potential impact on therapeutic efficacy. In CC in the present study, CDK1 correlated with CD8 naïve, effector memory, CD4 T, and NKT cells. Likewise, a post-hoc analysis revealed a substantial link between CDK1 and OC B, effector memory, and CD4 + T cells. Immune cells are pivotal in the tumor microenvironment (TME), significantly influencing tumor development and form while concurrently acting as tumor suppressors or promoters 35. Currently, conventional therapy mainly includes surgery, radiation therapy, and chemotherapy, while immunotherapy can avoid many shortcomings of conventional therapy. A very specific and effective aspect of CC treatment is that it involves effector memory cells. In women with locally advanced CC, ipilimumab, a checkpoint inhibitor, exhibits immunomodulatory activity when used in conjunction with chemoradiotherapy (CRT), leading to increased numbers of effector and central memory T cells 36. One important aspect that should not be disregarded in the TME process of OC is the aging of T cells. The main reason for this aging process is the deactivation and decrease in the number of T cells caused by the programmed cell death protein 1/programmed cell death ligand 1 (PD-1/PD-L1) pathway, which makes it easier for cancer to evade the immune system. Increased CD154 expression and cytokine release can effectively activate CD4+ tumor-infiltrating lymphocytes (TILs) during anti-PD-1 therapy, which promotes dendritic cell development and enhances CD8+ TIL multiplication. Immunosuppressive treatment may improve the body's ability to recuperate from chemotherapy while also suppressing its detrimental effects 37,38. However, no evidence exists to suggest that using a single drug—anti-PD-1 /PD-L1 antibodies—increases survival in patients with OC. In contrast, the checkpoint molecule B7-H3 is associated with decreased T-cell function, is substantially expressed in cancer, and is associated with tumor invasiveness. Therefore, immune system regulation offers a novel strategy when chemotherapy is inefficient or when OC is drug-resistant. This component is critical for the course of treatment in patients with a high risk of metastasis and recurrence 39. Cellular metabolism is not just a byproduct of cellular activities; it actively influences cell functions. This metabolic state is vital for adapting to the tumor microenvironment's challenges, like limited nutrients and suppressive signals. Memory T cells boost these metabolic processes, allowing them to self-renew and quickly respond to new threats 40. Thymic progenitor cells originate in the thymus gland, whereas T cells originate in the bone marrow. These cells can be generically categorized as NKT cells, rear gamma delta group T cells, and CD4+ and CD8+αβT cells. The functions of naïve CD4+ and CD8+ T cells include clonal proliferation, differentiation, and activation, which allow them to infect cells. They also produce cytokines and regulate immune responses, including TNF and interleukins 41. These lymphocytes can directly attack target cells or release a variety of inflammatory mediators to induce anti-inflammatory effects. Some T cells undergo memory cell transformation, enabling them to respond quickly to recurrent lesions. Additionally, they can provide effective and long-lasting protection 42. CD4+ and CD8+ T cells of cervical origin are mainly effector memory T cells (CCR7-/CD45RA-) 43. The T helper-1-like CD4+ population is linked to tumorigenicity and inflammation, initially releasing cytokines before transitioning to a cytotoxic state. Subsequently, activated CD8+ T-cell populations gradually infiltrate intraepithelial and cytotoxic tissues 44. Treatment decisions for female-specific tumors, such as ovarian, breast, endometrial, and cervical cancers, depend on the accurate collection and processing of tissue samples as well as IHC and molecular data. Strong antitumor immunity and immunotherapy critically depend on immune cell function. In the present study, we extensively investigated immune infiltration in CC and OC, as immunotherapy has not yet produced the anticipated results despite the possibility that OC and CC will respond to this treatment due to endogenous immune responses at the molecular or immune cell levels.

Nonetheless, there are certain constraints associated with bioinformatic analysis. Initially, the effectiveness of bioinformatics analysis heavily relies on the quality of the initial data; further, our developed model relies solely on the TCGA database. Consequently, data pertaining to race, region, nation, or ethnicity are essential to enhance the widespread relevance of prognostic models and biomarkers. Furthermore, the intricate nature of biological systems frequently leads to oversimplifications when bioinformatics analyses are performed, potentially resulting in imprecise modeling or forecasts. Tackling these challenges necessitates the use of multicenter, extensive clinical cohort studies and the incorporation of dietary patterns and medications as adjustment variables to improve the validation of upcoming prognostic models and biomarkers. Our research offers valuable insights for both preventing and treating OC and CC through the lens of external metabolic processes.

In summary, in the present study, we successfully identified a link between CDK1 and the development of CC and OC. CDK1 may be regarded as a crucial biomarker for induced CC and OC. The insights from our study will offer valuable references for subsequent investigations.

## Conclusions

Generally, the results show that CDK1 can effectively distinguish between samples from patients with CC and OC and normal controls, demonstrating to be a useful diagnostic marker. The study used bioinformatics methods to predict the potential functional role of CDK1 and detected the expression of CDK1 in CC and OC by immunohistochemical assay, which investigated their plausible involvement in their underlying mechanisms. The biological role and molecular processes of CDK1 in promoting the pathogenesis of CC and OC will also be the subject of future research.

## Figures and Tables

**Table 1:** Clinical parameters of patients with ovarian cancer.

Category	Subcategory	N = 90	Percent
Age, years	Median	52	
	Range	32-75	
Tumor size	≤ 5 cm	14	16%
	> 5 cm	14	16%
	NA	62	69%
TMN stage	IIIA	36	40%
	IIIC	52	58%
	IV	2	2%
Histology	High-grade serous carcinoma	86	96%
	High-grade serous carcinoma with transitional cell carcinoma	2	2%
	High-grade serous carcinoma with clear cell carcinoma	2	2%
Pelvic node involvement	Positive	26	29%
	Negative	64	71%

TNM, tumor node metastasis classification

**Table 2:** Clinical parameters of patients with cervical cancer.

Category	Subcategory	N = 30	Percent
Age, years	Median	51	
	Range	37-63	
Tumor size	≤ 5 cm	19	63%
	> 5 cm	1	3%
	NA	10	33%
Histology	Squamous	30	100%
Pelvic node involvement	Positive	9	30%
	Negative	21	70%

NA, not applicable as data not available. FIGO, International Federation of Gynaecology and Obstetrics

**Table 3:** Association between CDK1 and cervical or ovarian cancer was analyzed by Mendelian randomization.

Outcome	Method	pval	OR	OR_lci95	OR_uci95
Ovarian cancer	Inverse variance weighted	0.039091281	0.781840118	0.618847007	0.987762668
Cervical cancer	Wald ratio	0.022061183	1.001425012	1.00020486	1.002646652

OR, odds ratio

## Abbreviations

CC: cervical cancer
CDK1: cyclin dependent kinase 1
CNV: copy number variation
GO: gene ontology
GCSA: Gene Set Cancer Analysis
GSVA: gene set variation analysis
ICGC: International Cancer Genome Consortium
IHC: immunohistochemistry
IV: instrumental variables
KEGG: Kyoto Encyclopedia of Genes and Genomes
OC: ovarian cancer
ROC: receiver operating characteristic
SNP: single nucleotide polymorphism
TCGA: The Cancer Genome Atlas

## References

[1] Janjua D, Thakur K, Aggarwal N, Chaudhary A, Yadav J, Chhokar A (2024). Prognostic and therapeutic potential of STAT3: Opportunities and challenges in targeting HPV-mediated cervical carcinogenesis. Crit Rev Oncol Hematol.

[2] Ouh YT, Kim HY, Yi KW, Lee NW, Kim HJ, Min KJ (2024). Enhancing Cervical Cancer Screening: Review of p16/Ki-67 Dual Staining as a Promising Triage Strategy. Diagnostics (Basel).

[3] Luvero D, Angioli R, Celoro F, Plotti F, Terranova C, Guzzo F (2024). Tailored Treatment Strategies in First Line Therapy for Ovarian Cancer Patients: A Critical Review of the Literature. Pharmaceuticals (Basel).

[4] Yeoh SA, Webb S, Phillips A, Li LSK, Kumar S (2024). Psychosocial interventions for ovarian cancer survivors: A systematic review. Psychooncology.

[5] Massacci G, Perfetto L, Sacco F (2023). The Cyclin-dependent kinase 1: more than a cell cycle regulator. Br J Cancer.

[6] Izadi S, Nikkhoo A, Hojjat-Farsangi M (2020). CDK1 in Breast Cancer: Implications for Theranostic Potential. Anticancer Agents Med Chem.

[7] Schwartz GK, O'Reilly E, Ilson D (2002). Phase I study of the cyclin-dependent kinase inhibitor flavopiridol in combination with paclitaxel in patients with advanced solid tumors. J Clin Oncol.

[8] Wang Q, Bode AM, Zhang T (2023). Targeting CDK1 in cancer: mechanisms and implications. NPJ Precis Oncol.

[9] Sutaji Z, Elias MH, Ahmad MF, Karim AKA, Abu MA (2022). A Systematic Review and Integrated Bioinformatic Analysis of Candidate Genes and Pathways in the Endometrium of Patients With Polycystic Ovary Syndrome During the Implantation Window. Front Endocrinol (Lausanne).

[10] Wei X, Deng W, Dong Z, Xie Z, Zhang J, Wang R (2022). Identification of Subtypes and a Delayed Graft Function Predictive Signature Based on Ferroptosis in Renal Ischemia-Reperfusion Injury. Front Cell Dev Biol.

[11] Zhang X, Xiao J, Fu X, Qin G, Yu M, Chen G (2022). Construction of a Two-Gene Immunogenomic-Related Prognostic Signature in Lung Squamous Cell Carcinoma. Front Mol Biosci.

[12] Recaldin T, Steinacher L, Gjeta B, Harter MF, Adam L, Kromer K (2024). Human organoids with an autologous tissue-resident immune compartment. Nature.

[13] Zhang Q, Zhang H, Xu Q (2024). Association of Chronic Obstructive Pulmonary Disease with Risk of Psychiatric Disorders: A Two-Sample Mendelian Randomization Study. Int J Chron Obstruct Pulmon Dis.

[14] Goh S, Thiyagarajan L, Dudding-Byth T, Mark P, Kirk EP (2024). A systematic review and pooled analysis of penetrance estimates of copy number variants associated with neurodevelopment. Genet Med.

[15] Wei E, Reisinger A, Li J, French LE, Clanner-Engelshofen B, Reinholz M (2022). Integration of scRNA-Seq and TCGA RNA-Seq to Analyze the Heterogeneity of HPV+ and HPV- Cervical Cancer Immune Cells and Establish Molecular Risk Models. Front Oncol.

[16] Njoku K, Abiola J, Russell J, Crosbie EJ (2020). Endometrial cancer prevention in high-risk women. Best Pract Res Clin Obstet Gynaecol.

[17] Horst KE, Modesitt SC (2016). Hormonal Contraceptives for Endometrial Cancer Prevention in Obese and High-Risk Women in Virginia. South Med J.

[18] Li J, Yang Z, Wang T, Li M, Wu X, Fu X (2024). Causal relationship between lipid-lowering drugs and ovarian cancer, cervical cancer: a drug target mendelian randomization study. BMC Cancer.

[19] Cao Z, Long X, Yuan L (2024). Associations between serum metabolites and female cancers: A bidirectional two-sample mendelian randomization study. J Steroid Biochem Mol Biol.

[20] Antonsen SL, Høgdall E, Christensen IJ, Lydolph M, Tabor A, Loft Jakobsen A (2013). HE4 and CA125 levels in the preoperative assessment of endometrial cancer patients: a prospective multicenter study (ENDOMET). Acta Obstet Gynecol Scand.

[21] Healthcare Engineering JO (2023). Retracted: Overexpressed RING Finger 44 Correlates with Poor Prognosis in Hepatocellular Carcinoma. J Healthc Eng.

[22] Huang Y, Fan Y, Zhao Z (2023). Inhibition of CDK1 by RO-3306 Exhibits Anti-Tumorigenic Effects in Ovarian Cancer Cells and a Transgenic Mouse Model of Ovarian Cancer. Int J Mol Sci.

[23] Li J, Zhi X, Shen X (2020). Depletion of UBE2C reduces ovarian cancer malignancy and reverses cisplatin resistance via downregulating CDK1. Biochem Biophys Res Commun.

[24] Cedeno-Rosario L, Honda D, Sunderland AM, Lewandowski MD, Taylor WR, Chadee DN (2022). Phosphorylation of mixed lineage kinase MLK3 by cyclin-dependent kinases CDK1 and CDK2 controls ovarian cancer cell division. J Biol Chem.

[25] Chen S, Chen X, Xiu YL, Sun KX, Zhao Y (2015). MicroRNA-490-3P targets CDK1 and inhibits ovarian epithelial carcinoma tumorigenesis and progression. Cancer Lett.

[26] Qiao L, Zheng J, Tian Y (2018). Regulator of chromatin condensation 1 abrogates the G1 cell cycle checkpoint via Cdk1 in human papillomavirus E7-expressing epithelium and cervical cancer cells. Cell Death Dis.

[27] Luo Y, Wu Y, Peng Y, Liu X, Bie J, Li S (2016). Systematic analysis to identify a key role of CDK1 in mediating gene interaction networks in cervical cancer development. Ir J Med Sci.

[28] Wu M, Mu C, Yang H, Wang Y, Ma P, Li S (2024). 8-Br-cGMP suppresses tumor progression through EGFR/PLC γ1 pathway in epithelial ovarian cancer. Mol Biol Rep.

[29] Gong M, Shen F, Li Y, Ming L, Hong L (2023). MLK4 as an immune marker and its correlation with immune infiltration in Cervical squamous cell carcinoma and endocervical adenocarcinoma (CESC). PLoS One.

[30] Craig O, Lee S, Pilcher C, Saoud R, Abdirahman S, Salazar C (2024). A new method for network bioinformatics identifies novel drug targets for mucinous ovarian carcinoma. NAR Genom Bioinform.

[31] Zhou Y, Xie Y, Luo Y, Wang S, Han Q, Liu Q (2024). SNAI2 enhances HPV-negative cervical cancer cell dormancy by modulating u-PAR expression and the activity of the ERK/p38 signaling pathway in vitro. Oncol Rep.

[32] Sánchez-Martínez C, Lallena MJ, Sanfeliciano SG, de Dios A (2019). Cyclin dependent kinase (CDK) inhibitors as anticancer drugs: Recent advances (2015-2019). Bioorg Med Chem Lett.

[33] Hayashi Y, Fujimura A, Kato K, Udagawa R, Hirota T, Kimura K (2018). Nucleolar integrity during interphase supports faithful Cdk1 activation and mitotic entry. Sci Adv.

[34] Timofeev O, Cizmecioglu O, Settele F, Kempf T, Hoffmann I (2010). Cdc25 phosphatases are required for timely assembly of CDK1-cyclin B at the G2/M transition. J Biol Chem.

[35] Locy H, de Mey S, de Mey W, De Ridder M, Thielemans K, Maenhout SK (2018). Immunomodulation of the Tumor Microenvironment: Turn Foe Into Friend. Front Immunol.

[36] Da Silva DM, Enserro DM, Mayadev JS, Skeate JG, Matsuo K, Pham HQ (2020). Immune Activation in Patients with Locally Advanced Cervical Cancer Treated with Ipilimumab Following Definitive Chemoradiation (GOG-9929). Clin Cancer Res.

[37] Zhao J, Wang Z, Tian Y, Ning J, Ye H (2024). T cell exhaustion and senescence for ovarian cancer immunotherapy. Semin Cancer Biol.

[38] Liu X, Hartman CL, Li L, Albert CJ, Si F, Gao A (2021). Reprogramming lipid metabolism prevents effector T cell senescence and enhances tumor immunotherapy. Sci Transl Med.

[39] Cai D, Li J, Liu D, Hong S, Qiao Q, Sun Q (2020). Tumor-expressed B7-H3 mediates the inhibition of antitumor T-cell functions in ovarian cancer insensitive to PD-1 blockade therapy. Cell Mol Immunol.

[40] Claiborne MD (2022). Manipulation of metabolic pathways to promote stem-like and memory T cell phenotypes for immunotherapy. Front Immunol.

[41] Yui MA, Rothenberg EV (2014). Developmental gene networks: a triathlon on the course to T cell identity. Nat Rev Immunol.

[42] Emmanuel T, Mistegård J, Bregnhøj A, Johansen C, Iversen L (2021). Tissue-Resident Memory T Cells in Skin Diseases: A Systematic Review. Int J Mol Sci.

[43] Posavad CM, Zhao L, Dong L, Jin L, Stevens CE, Magaret AS (2017). Enrichment of herpes simplex virus type 2 (HSV-2) reactive mucosal T cells in the human female genital tract. Mucosal Immunol.

[44] Zhou M, Liu ZL, Liu JY, Wang XB (2024). Tedizolid phosphate alleviates DSS-induced ulcerative colitis by inhibiting senescence of cell and colon tissue through activating AMPK signaling pathway. Int Immunopharmacol.

